# Aging-related metabolic dysregulation in osteoporosis: mechanisms and therapeutic strategies

**DOI:** 10.3389/fragi.2026.1886673

**Published:** 2026-07-27

**Authors:** Ruifeng Bai, Zishuai Huang, Xuan Tian, Yikai Liu, Yan Wang, Yu Su, Minjuan Li, Cheng Cheng, Jun Wu, Yejun Zha, Shuai Lu

**Affiliations:** 1 Department of Clinical Laboratory, Beijing Jishuitan Hospital, Capital Medical University, Beijing, China; 2 Department of Orthopedic Trauma, Beijing Jishuitan Hospital, Capital Medical University, Beijing, China; 3 Department of Vascular Surgery, Beijing Jishuitan Hospital, Capital Medical University, Beijing, China; 4 Department of Osteoporosis, Beijing Jishuitan Hospital, Capital Medical University, Beijing, China; 5 Beijing Research Institute of Traumatology and Orthopaedics, Beijing, China

**Keywords:** cellular senescence, metabolic dysfunction, Bone remodeling, multitarget therapy, senile osteoporosis, skeletal aging

## Abstract

**Purpose of review:**

This review aims to summarize recent advances in the mechanistic understanding of senile osteoporosis, with particular focus on the interconnected roles of cellular senescence, metabolic dysfunction, and systemic homeostatic imbalance in age-related skeletal degeneration.

**Recent findings:**

Emerging evidence indicates that senile osteoporosis is not driven solely by age-related hormonal decline, but by a complex network of biological processes involving senescence of bone marrow mesenchymal stem cells, accumulation of the senescence-associated secretory phenotype, mitochondrial dysfunction, oxidative stress, chronic low-grade inflammation, and disturbances in glucose and lipid metabolism. These alterations disrupt bone remodeling through key signaling pathways, including RANKL/OPG, Wnt/β-catenin, AMPK/SIRT1, NF-κB, and PI3K/Akt/mTOR. Together, these mechanisms impair osteogenesis, enhance osteoclastogenesis, deteriorate bone microarchitecture, and increase skeletal fragility. This broader pathophysiological framework may explain why conventional antiresorptive therapies, although effective in reducing bone resorption, often fail to fully restore the structural and functional deficits of the aging skeleton.

**Summary:**

Senile osteoporosis should be viewed as a systemic aging-related disorder involving both deterioration of the local bone microenvironment and whole-body metabolic dysregulation. Current evidence-based pharmacological treatments, including bisphosphonates, denosumab, teriparatide, abaloparatide, and romosozumab, remain central to fracture prevention and bone mass preservation. However, these interventions do not fully reverse the biological processes of skeletal aging. Emerging strategies targeting cellular senescence, the senescence-associated secretory phenotype, mitochondrial dysfunction, oxidative stress, nutrient-sensing pathways, and gut microbiota are under active investigation and may complement established therapies in the future. A clearer distinction between approved anti-osteoporotic drugs and experimental geroscience-based interventions is essential for translating mechanistic insights into clinically meaningful treatment strategies.

## 1 Introduction

Osteoporosis is a common systemic skeletal disease characterized by reduced bone mass, deterioration of bone microarchitecture, and increased fracture risk. Its prevalence increases with age, making it an important health issue in the elderly population (Wang G. et al., 2025; Ali et al., 2022). Age-related osteoporosis is closely linked to changes in systemic metabolism. It is not simply a local consequence of bone aging, but is also influenced by broader alterations in energy, lipid, and glucose metabolism, chronic low-grade inflammation, and hormonal regulation (Zhao Y. et al., 2025; Imerb et al., 2020). These changes disturb bone remodeling and gradually shift the balance toward bone loss. One example is the age-related expansion of bone marrow adipose tissue (BMAT), which is often accompanied by reduced osteogenic differentiation. BMAT accumulation has been implicated in increased bone fragility, particularly in obesity and type 2 diabetes (T2D) (Ali et al., 2022). Moreover, bone marrow adipocyte-derived factors may induce osteoblasts to acquire adipocyte-like features, further contributing to age-related bone loss (Clabaut et al., 2021).

Traditionally, age-related osteoporosis has been mainly linked to declining sex hormone levels. However, recent studies suggest that changes in the bone marrow microenvironment, senescent cell accumulation, mitochondrial dysfunction, and systemic metabolic disorders also contribute importantly to bone loss (Zhao Y. et al., 2025; Zhong et al., 2024). Cellular senescence, especially the senescence-associated secretory phenotype (SASP), promotes chronic low-grade inflammation in bone, often referred to as inflammaging, and disrupts bone homeostasis (Zhao Y. et al., 2025; Cheng et al., 2025). Senescent bone marrow mesenchymal stem cells (BMSCs) show reduced osteogenic potential, while osteoclast activity may increase (Zhong et al., 2024; Yoon et al., 2024). In aged mice, accumulation of histone deacetylase 6 (HDAC6) in bone marrow stromal cells suppresses osteogenic differentiation by reducing histone acetylation at the Runx2 promoter (Ma et al., 2021). Immunosenescence may further enhance osteoclastogenesis and impair the osteogenic microenvironment through SASP-related factors, contributing to immune–bone imbalance (Cheng et al., 2025).

Although conventional anti-osteoporotic therapies effectively reduce fracture risk, they primarily target bone remodeling rather than the upstream biological drivers of skeletal aging. Antiresorptive agents such as bisphosphonates and denosumab inhibit osteoclast-mediated bone resorption, whereas anabolic agents such as teriparatide, abaloparatide, and romosozumab promote bone formation or rebalance bone remodeling. These treatments are clinically established and remain the foundation of osteoporosis management. In contrast, interventions targeting cellular senescence, mitochondrial dysfunction, inflammaging, altered nutrient sensing, and gut microbiota are still largely investigational in the context of osteoporosis (Cheng et al., 2025; Ulivieri et al., 2025; Roux and Briot, 2020). Therefore, this review summarizes the main mechanisms linking metabolic imbalance to osteoporosis, outlines related signaling pathways, and discusses therapeutic strategies based on metabolic regulation and senescence intervention.

## 2 Imbalance in bone remodeling and aging of the bone marrow microenvironment

### 2.1 Effects of aging on the function of the basic multicellular unit in bone remodeling

During aging, the bone remodeling unit (BMU) undergoes functional changes that contribute to bone loss and osteoporosis. Bone BMSCs, as osteoblast precursors, show reduced proliferation, self-renewal, and osteogenic differentiation with age. Under senescent conditions, BMSCs are less likely to differentiate into osteoblasts and more likely to undergo adipogenic differentiation, leading to reduced bone formation and increased bone marrow fat accumulation (Fang et al., 2022). This shift is related to age-associated epigenetic changes, oxidative stress, and chronic low-grade inflammation, which together contribute to impaired bone formation in the aged skeleton (Liang et al., 2026). Osteoclast activity also changes with aging. Although the number of osteoclast precursors may not increase markedly, their differentiation and resorptive activity can be enhanced under chronic inflammatory stimulation (Kitazawa et al., 2025). Increased levels of pro-inflammatory cytokines, such as tumor necrosis factor-α (TNF-α) and interleukin-6 (IL-6), can upregulate receptor activator of nuclear factor-κB ligand (RANKL), thereby promoting osteoclastogenesis and bone resorption (Shin et al., 2024). When bone resorption exceeds bone formation, progressive bone loss occurs, leading to trabecular thinning, cortical loss, and deterioration of bone microarchitecture (Ramchand and Seeman, 2020).

Osteocytes, which are embedded in the bone matrix, are important regulators of mechanosensing and bone remodeling. Aging impairs the osteocyte network, with increased apoptosis, reduced autophagy, and weakened cell–cell communication. These changes lead to impaired mechanotransduction and altered paracrine signaling (Huang et al., 2026). Senescent osteocytes also produce more sclerostin (SOST) and less osteoprotegerin (OPG), which suppresses osteoblast activity and favors osteoclastogenesis (Kitazawa et al., 2025; Jiao et al., 2023). In addition, their reduced sensitivity to mechanical loading limits adaptive bone remodeling and accelerates age-related bone loss (Jiao et al., 2023; Liang et al., 2024). Osteocyte-derived RANKL may also regulate cortical bone remodeling, although its role during aging requires further study (Kitazawa et al., 2025). Overall, aging disrupts BMU function by reducing BMSC osteogenic potential, increasing osteoclast-mediated resorption, and impairing osteocyte function. These changes shift bone remodeling toward net bone loss and contribute to senile osteoporosis. Targeting stem cell fate, excessive resorption, and osteocyte dysfunction may therefore provide useful strategies for its prevention and treatment.

### 2.2 Pathological significance of bone marrow adiposity

Bone marrow adiposity is a common feature of aging and metabolic bone disorders and contributes to osteoporosis. With age, adipose tissue accumulates in the bone marrow cavity, partly replacing hematopoietic and osteogenic niches (Yu et al., 2023). This reduces the space needed for osteoblast function and creates a local environment that is unfavorable for bone formation through paracrine signaling (Yu et al., 2023; Beekman et al., 2023; Pachón-Peña and Bredella, 2022; Niu et al., 2024). Bone marrow adipose tissue is metabolically active. It secretes adipokines, lipid mediators, and inflammatory factors, including leptin, adiponectin, resistin, tumor necrosis factor-α (TNF-α), and interleukin-6 (IL-6) (Pachón-Peña and Bredella, 2022; Niu et al., 2024; Aaron et al., 2022). These factors can inhibit osteoblast differentiation and promote osteoclastogenesis through the receptor activator of nuclear factor-κB ligand/osteoprotegerin (RANKL/OPG) axis, leading to increased bone resorption and bone loss (Aaron et al., 2022). In postmenopausal osteoporosis models, marrow fat expansion is associated with reduced bone formation and enhanced bone resorption (Li J. et al., 2024). Thus, bone marrow adiposity is not merely a space-filling change, but an active driver of bone loss.

A key mechanism is the altered differentiation of BMSCs. Osteoblasts and marrow adipocytes share the same progenitors, and aging, estrogen deficiency, and inflammation shift BMSC fate from osteogenesis toward adipogenesis (Xu et al., 2024a; Xiao et al., 2026). This shift is marked by activation of peroxisome proliferator-activated receptor gamma (PPARγ) and suppression of osteogenic factors such as Runt-related transcription factor 2 (Runx2) and Osterix (Xu et al., 2024a; Rino et al., 2024). As a result, adipocytes increase, osteoblast formation declines, and bone formation is reduced (Beekman et al., 2023; Li J. et al., 2024). Overall, bone marrow adiposity promotes osteoporosis by reshaping the marrow environment, enhancing inflammation, and shifting BMSC differentiation toward adipogenesis. Targeting this imbalance may help prevent age-related bone loss.

## 3 The driving role of cellular senescence and the SASP

### 3.1 Accumulation of senescent cells in the bone microenvironment

During aging, osteoblasts, osteocytes, and BMSCs gradually enter cellular senescence, a state of irreversible cell-cycle arrest (Khosla et al., 2022). This process involves not only reduced proliferative capacity, but also metabolic changes, epigenetic alterations, and functional decline (Gerosa et al., 2023). For instance, in senescent osteoblasts, the level of methyltransferase 3 (METTL3)-mediated m6A RNA methylation is significantly reduced, thereby weakening its stabilizing effect on SIRT1 mRNA, which in turn exacerbates cellular senescence and suppresses proliferation (Chen et al., 2025). Senescent BMSCs also show impaired Golgi function, leading to reduced self-renewal and osteogenic differentiation (Liu et al., 2025). These changes contribute to skeletal aging and osteoporosis. The accumulation of senescent cells in the bone microenvironment is closely linked to age-related bone loss and deterioration of bone structure (Doolittle et al., 2021). In osteoporosis models, senescence-associated immune cells such as bone-resident macrophages also increase, which further promotes local inflammation (Nishida et al., 2024). Thus, the accumulation of senescent cells in the bone microenvironment, by disrupting intrinsic cellular homeostatic mechanisms, provides a cellular basis for senescence-related dysregulation of bone metabolism.

Senescent cells are active rather than inert. They secrete the SASP, which includes cytokines, chemokines, growth factors, and matrix metalloproteinases (MMPs) (Xu et al., 2021; Wang Y. et al., 2021). In bone, senescent osteocytes are an important source of SASP. Radiation-induced senescent osteocyte-like MLO-Y4 cells increase the release of IL-1α, IL-6, and MMP-3 (Xu et al., 2021). These factors act on neighboring cells through paracrine signaling. SASP from senescent osteocytes can suppress the osteogenic and adipogenic differentiation of BMSCs (Xu et al., 2021). It also promotes osteoclastogenesis and inhibits osteoblast function through factors such as RANKL, leading to increased bone resorption and reduced bone formation (Wang Y. et al., 2021; Wang Y. et al., 2023). In a breast cancer bone metastasis model, tumor-induced osteocyte senescence and SASP were identified as early events in osteolytic bone destruction (Kaur et al., 2024). In addition, SASP also contributes to chronic low-grade inflammation, or inflammaging, which further drives senescence and accelerates bone loss (Gerosa et al., 2023). Overall, senescent cells and SASP are key links between aging and bone dysfunction and play an important role in osteoporosis.

### 3.2 The detrimental effects of SASP on bone homeostasis

The senescence-associated secretory phenotype refers to a complex array of bioactive molecules secreted by senescent cells, which act through paracrine and endocrine mechanisms to establish and sustain a persistent pro-inflammatory state in the bone microenvironment, thereby directly disrupting bone homeostasis (Fan et al., 2024). SASP components include a variety of pro-inflammatory cytokines, chemokines, and proteases that can directly modulate the function of key bone-resident cells. For example, classical SASP factors such as interleukin-6 (IL-6) and tumor necrosis factor-α (TNF-α) can effectively promote the activation and differentiation of osteoclast precursors, thereby enhancing osteoclastic bone-resorptive activity (Chang et al., 2022). Meanwhile, these pro-inflammatory mediators exert pronounced inhibitory effects on osteoblast differentiation and function. Studies have shown that SASP factors including IL-1α, IL-1β, and CCL2 can activate signaling pathways such as NF-κB, thereby interfering with the expression of the osteogenic master transcription factor Runx2, suppressing osteoblast mineralization capacity, and ultimately reducing bone formation (Kim et al., 2022; Lee et al., 2025). This dual effect of promoting bone resorption while inhibiting bone formation contributes to a negative bone metabolic balance, representing a central mechanism in the onset and progression of osteoporosis.In addition, SASP is not restricted to a single cell type; senescent osteoblasts, osteoclasts, BMSCs, and osteocytes can all produce deleterious SASP factors, thereby forming a complex regulatory network that cooperatively disrupts bone homeostasis (Huo et al., 2024; Yang D. et al., 2025).

More critically, SASP can also induce senescence in neighboring otherwise healthy cells through paracrine signaling, forming a self-amplifying vicious cycle that accelerates the deterioration and dysfunction of the entire bone microenvironment (Xiong et al., 2025). This “senescence propagation” effect enables the rapid spread of senescent phenotypes within bone tissue. For instance, SASP factors secreted by senescent osteocytes or BMSCs can be taken up by adjacent BMSCs, activating the DNA damage response and the p53/p21 signaling axis, thereby inducing senescence and triggering the production of new SASP factors (Xu et al., 2021). This cascade markedly amplifies senescence- and inflammation-related signals.In the senescent bone microenvironment, accumulated SASP factors further recruit and activate immune cells, such as macrophages, thereby exacerbating local inflammation and establishing a chronic, low-grade inflammatory milieu known as “inflammaging” (Wang Z. et al., 2023). Such persistent inflammation is not only a major driver of bone loss, but also a key mechanistic bridge linking aging, chronic inflammation, and age-related disorders such as osteoporosis (Chandra and Rajawat, 2021; Zhu et al., 2022). Therefore, through both direct impairment of bone cell function and indirect propagation of senescence, SASP serves as a central pathological driver of age-associated bone loss and osteoporosis progressio (Wang H. et al., 2025; Wang T. et al., 2021).

## 4 Central role of oxidative stress and mitochondrial dysfunction

### 4.1 Age-related accumulation of reactive oxygen species

Mitochondrial dysfunction is a major source of excessive reactive oxygen species (ROS) during aging and is closely related to osteoporosis. With age, electron transport chain efficiency declines, mitochondrial DNA mutations accumulate, and antioxidant systems such as SOD and GSH weaken, all of which increase ROS production (Zia et al., 2022). This phenomenon is particularly evident in the skeletal system. In mesenchymal progenitor cells derived from aged mice, both the expression and activity of mitochondrial superoxide dismutase 2 (SOD2) are markedly reduced, accompanied by increased mitochondrial ROS generation (Schoppa et al., 2022). In osteoblast-lineage-specific Sod2 knockout mice, bone loss is accompanied by reduced osteoblast activity, increased marrow adiposity, and enhanced osteoclast activity, showing that mitochondrial ROS disrupts progenitor differentiation and promotes osteoblast senescence (Schoppa et al., 2022). Aging osteoblasts also show metabolic changes, including mitochondrial dysfunction, reduced metabolic flexibility, and lipid droplet accumulation, which further increase oxidative stress and contribute to bone loss (Nandy et al., 2024). In addition to intrinsic mitochondrial dysfunction, age-related endocrine changes may further amplify oxidative stress in bone.Vitamin D deficiency is common in older adults and contributes to impaired calcium-phosphate homeostasis, secondary hyperparathyroidism, reduced muscle function, and increased fracture risk. Recent experimental evidence also suggests that vitamin D insufficiency may directly promote oxidative stress and cellular senescence in bone-related cells. Mechanistically, vitamin D deficiency can increase ROS accumulation, induce DNA damage, and activate the p16-dependent senescence pathway, thereby impairing osteoblast function and bone formation (Qiao et al., 2025). Thus, vitamin D status may represent an important systemic modifier linking aging, redox imbalance, and skeletal fragility.

**FIGURE 1:**
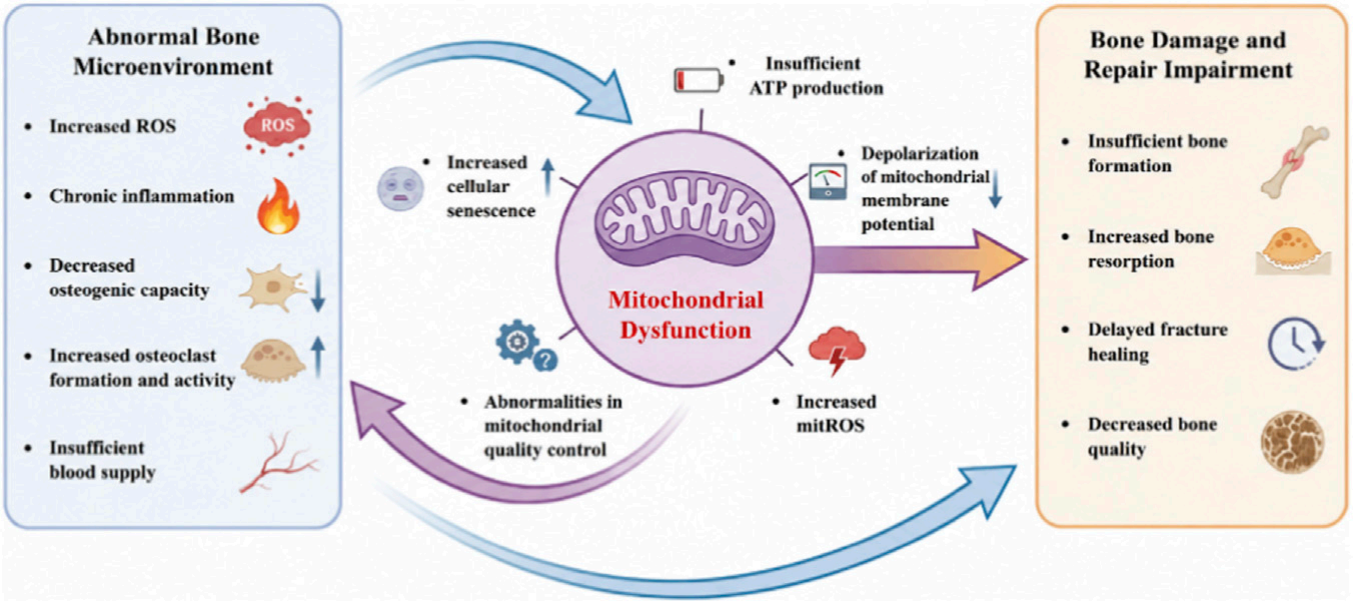
Oxidative stress-driven osteoblast and osteocyte dysfunction in aging-related osteoporosis.

Excessive ROS production damages intracellular macromolecules, including proteins, lipids, and DNA, and thereby compromises the survival and function of bone-forming cells. Oxidative injury can induce protein carbonylation, lipid peroxidation, mitochondrial membrane depolarization, and oxidative DNA lesions such as 8-hydroxy-2′-deoxyguanosine (8-OHdG) (Qiao et al., 2025). In osteoblasts and osteocytes, these alterations activate stress-response and apoptotic pathways, reduce osteogenic activity, increase sclerostin expression, and ultimately disturb bone remodeling homeostasis. Such oxidative injury is particularly detrimental to bone cells. For example, advanced oxidation protein products (AOPPs), recognized as markers of oxidative stress, accumulate with age and stimulate ROS production through activation of nicotinamide adenine dinucleotide phosphate (NADPH) oxidase (Zeng et al., 2021). In osteoblasts, AOPP-induced ROS accumulation suppresses sirtuin 1 (SIRT1) expression, leading to upregulation of sclerostin and thereby exacerbating age-related bone loss (Zeng et al., 2021). AOPPs can also cause mitochondrial membrane depolarization, further increase mitochondrial ROS, and promote osteoblast apoptosis (Li et al., 2023). In addition, age-related declines in melatonin reduce antioxidant capacity, increase osteoclastogenesis, and lower bone mass (Wu et al., 2024). Melatonin supplementation has been shown to markedly inhibit receptor activator of nuclear factor kappa-B ligand (RANKL)-induced osteoclastogenesis and reduce intracellular ROS levels (Wu et al., 2024). In aged osteoblasts, reduced motif-containing 16 (TRIM16) weakens the antioxidant response by lowering 2-related factor 2 (Nrf2), heme oxygenase-1 (HO-1), and SOD1 expression, leading to higher ROS and impaired osteogenic differentiation (Li K. et al., 2024). Taken together, these findings demonstrate that excessive \ROS accumulation during aging poses a substantial threat to the survival and function of osteoblasts and osteocytes by directly damaging cellular components and activating apoptotic signaling pathways. This process represents a major mechanism underlying reduced bone formation and the initiation and progression of osteoporosis (Figure 1).

### 4.2 Oxidative stress disrupts bone metabolic signaling pathways

Excessive accumulation of reactive oxygen species (ROS) during aging constitutes a critical mechanism driving the initiation and progression of osteoporosis by interfering with key signaling pathways involved in bone metabolism. On the one hand, ROS can directly activate signaling cascades that promote osteoclast differentiation. It has been shown that ROS activate the nuclear factor-kappa B (NF-κB) and mitogen-activated protein kinase (MAPK) pathways, both of which are major downstream signals required for receptor activator of nuclear factor kappa-B ligand (RANKL)-induced osteoclastogenesis (Li et al., 2020). For example, cinnamon tannin B-1 (CB-1) suppresses osteoclast formation and prevents ovariectomy-induced osteoporosis by inhibiting ROS generation and thereby blocking NF-κB pathway activation (Li et al., 2020). Another study demonstrated that SIS3 inhibits osteoclast differentiation and maturation by suppressing Smad3 phosphorylation, downregulating Nox4-dependent ROS production, and subsequently blocking MAPK phosphorylation (Pan et al., 2022). Together, these findings indicate that ROS-mediated activation of the NF-κB and MAPK pathways is a key event promoting enhanced bone resorption.On the other hand, excessive ROS exerts inhibitory effects on osteogenic signaling pathways. Under conditions of aging or estrogen deficiency, ROS accumulation disrupts the Wnt/β-catenin signaling pathway. In aged mice, high ROS levels have been shown to shift β-catenin-driven transcription away from TCF4 toward FOXO1-mediated transcription, thereby suppressing canonical Wnt/β-catenin signaling and reducing osteoblastic activity (Xiong et al., 2022). Similarly, in a mouse model with conditional deletion of MACF1, which mimics premature aging, chronic oxidative stress enhances the interaction between FoxO1 and β-catenin, competitively weakening β-catenin binding to TCF7 and thereby attenuating TCF7/β-catenin pathway activity and impairing osteoblast differentiation (Su et al., 2026). In addition, ROS negatively regulates the bone morphogenetic protein (BMP)/Smad pathway. It has been reported that excessive activation of the mitochondrial calcium uniporter (MCU) leads to mitochondrial calcium overload and ROS accumulation, which in turn negatively regulates BMP/Smad signaling and suppresses osteogenic differentiation (Li et al., 2025). Therefore, oxidative stress fundamentally disrupts the balance of bone remodeling by exerting a dual effect on bone metabolism signaling, namely, activation of osteoclastogenic pathways and inhibition of osteogenic pathways, and thus represents a central pathological basis of age-related bone loss (Shinohara et al., 2025).

The endogenous antioxidant defense system is essential for maintaining redox homeostasis and preserving skeletal health; however, its function is broadly compromised during aging, rendering it insufficient to counteract the progressively intensified oxidative stress and thereby exacerbating bone metabolic imbalance. Nuclear factor erythroid 2-related factor 2 (Nrf2) is a master regulator of the cellular antioxidant response. Upon dissociation from Kelch-like ECH-associated protein 1 (Keap1), Nrf2 translocates into the nucleus and initiates the transcription of downstream antioxidant genes, including heme oxygenase-1 (HO-1) (Lavhale et al., 2025). Nevertheless, the Nrf2/HO-1 pathway is impaired in the aging state. In a naturally aged NOD/SCID mouse model of osteoporosis, the expression of Nrf2 in bone tissue was significantly reduced with increasing age (Ma Z. et al., 2024). Activation of the Nrf2 pathway has been demonstrated to be an effective strategy for improving bone metabolism. For instance, natural plant-derived compounds such as curcumin and icariin B have been shown to ameliorate bone metabolic imbalance through modulation of the Nrf2/HO-1 pathway (Xiang et al., 2024). Another important antioxidant regulatory axis is the SIRT1/PGC-1α pathway. SIRT1 is a nicotinamide adenine dinucleotide (NAD+)-dependent deacetylase with anti-aging and antioxidant properties, and it activates PGC-1α to jointly maintain mitochondrial function and redox homeostasis (Zhao H. et al., 2025). Studies have shown that melatonin enhances the resistance of BMMSCs to oxidative stress-induced senescence by activating SIRT1, thereby preserving their osteogenic potential (Chen et al., 2022). Similarly, the novel drug eldecalcitol (ED-71) has been reported to alleviate macrophage senescence and improve osteoporosis through the SIRT1/PGC-1α signaling axis (Fu et al., 2026). In addition, the anti-osteoporotic effects of spermidine (SPD) have also been associated with activation of the SIRT1/SOD2 signaling pathway and enhancement of antioxidant capacity (Du et al., 2025). The forkhead box O (FOXO) family of transcription factors, particularly FOXO1 and FOXO3, also plays important roles in responding to oxidative stress and regulating bone cell fate (Su et al., 2026). Under aging conditions, FOXO1 competes with β-catenin for binding, thereby suppressing Wnt signaling-driven osteogenesis (Xiong et al., 2022). Taken together, aging is accompanied by functional decline of key endogenous antioxidant defense systems, including Nrf2/HO-1, SIRT1/PGC-1α, and FOXO, which compromises the ability to effectively eliminate excessive ROS.This weakened defense capacity sustains a persistently oxidative microenvironment in bone tissue, further amplifying the inhibitory effects of ROS on osteogenic signaling and its stimulatory effects on osteoclastogenic pathways, ultimately leading to a severe imbalance between bone formation and bone resorption and accelerating the progression of osteoporosis. Therefore, targeting and enhancing the function of these antioxidant pathways represents a promising therapeutic strategy for age-related osteoporosis (Table 1).

**TABLE 1:** Major ROS-associated signaling pathways and their regulatory agents in age-related osteoporosis.

Pathway	Mechanism	Agents
NF-κB/MAPK (Li et al., 2020)	ROS activates NF-κB and MAPK signaling, promoting RANKL-induced osteoclast differentiation and maturation	CB-1: inhibits ROS generation and blocks NF-κB activation
Smad3/Nox4/MAPK (Pan et al., 2022)	SIS3 suppresses osteoclast differentiation by inhibiting Smad3 phosphorylation, reducing Nox4-dependent ROS, and blocking MAPK phosphorylation	SIS3
Wnt/β-catenin (Xiong et al., 2022; Su et al., 2026)	High ROS shifts β-catenin-dependent transcription from TCF4/TCF7 to FOXO1, weakening Wnt/β-catenin signaling and osteogenesis	Curcumin, icariin B: indirectly improve osteogenesis *via* antioxidant pathways
BMP/Smad (Li et al., 2025)	Mitochondrial calcium overload and ROS accumulation negatively regulate BMP/Smad signaling and suppress osteogenic differentiation	Curcumin
Nrf2/HO-1 (Lavhale et al., 2025; Ma et al., 2024a)	Nrf2 induces antioxidant genes such as HO-1, but this pathway is impaired during aging, reducing antioxidant defense	Curcumin, icariin B
SIRT1/PGC-1α (Zhao et al., 2025b; Chen et al., 2022; Fu et al., 2026)	SIRT1 and PGC-1α maintain mitochondrial function and redox homeostasis; activation helps reduce BMMSC or macrophage senescence	Melatonin, ED-71, spermidine (SPD)
SIRT1/SOD2 (Du et al., 2025)	Enhances antioxidant capacity, reduces ROS accumulation, and improves bone metabolism	Spermidine (SPD)
FOXO1/FOXO3 (Xiong et al., 2022; Su et al., 2026)	FOXO family participates in oxidative stress responses; FOXO1 can also compete with β-catenin binding and suppress Wnt-mediated osteogenesis	Melatonin: enhances antioxidant response *via* SIRT1-related mechanisms

Entries include both established osteoporosis therapies and investigational agents. Natural products and other bioactive compounds listed here are supported mainly by preclinical evidence and should not be interpreted as approved treatments for osteoporosis.

## 5 Chronic low-grade inflammation and “inflammaging”

### 5.1 Characteristics of inflammaging

Aging is accompanied by immune dysregulation, characterized by excessive activation of innate immunity and progressive decline of adaptive immunity, collectively resulting in a systemic, chronic, low-grade inflammatory state known as “inflammaging” (Ponti et al., 2020). Rather than being triggered by acute infection, this represents a persistent sterile inflammatory milieu and is regarded as a key risk factor in the development of multiple age-related diseases (Zhou Y. et al., 2020). Aging is accompanied by a persistent, low-grade inflammatory state, commonly referred to as inflammaging.This condition is characterized by increased circulating levels of inflammatory mediators, including interleukin-6, tumor necrosis factor-α, interleukin-1β, and C-reactive protein. Rather than acting as isolated biomarkers, these mediators participate in a broad network of age-related tissue dysfunction and have been associated with frailty, cardiovascular disease, chronic kidney disease, and skeletal deterioration (Adamstein et al., 2023; Agboola et al., 2026). In the skeletal system, inflammatory cytokines promote osteoclastogenesis mainly by increasing RANKL expression and enhancing osteoclast precursor differentiation, while simultaneously suppressing osteoblast differentiation and function (Xu et al., 2024b).

Importantly, inflammaging is not limited to systemic circulation. Local inflammatory changes within the bone marrow microenvironment also contribute to age-related bone loss. Senescent osteocytes, osteoblast-lineage cells, bone marrow stromal cells, immune cells, and adipocytes can secrete components of the SASP, including IL-6, IL-1β, TNF-α, MCP-1, matrix metalloproteinases, and other pro-inflammatory factors. These local mediators alter osteoblast–osteoclast coupling, promote marrow adiposity, impair osteogenic differentiation of bone marrow mesenchymal stem cells, and enhance osteoclast activity (Xu et al., 2024b; Ma H. et al., 2024). Therefore, both systemic inflammaging and local bone marrow inflammation should be considered key contributors to the pathogenesis of age-related osteoporosis (Mazzaferro et al., 2020). This persistent low-grade inflammation disrupts bone cell activity and calcium homeostasis within bone tissue, ultimately leading to reduced bone mass and increased skeletal fragility.

The maintenance of inflammaging involves the interplay of multiple complex factors. Among them, the accumulation of senescent cells and their SASP play a central role (Zhou Y. et al., 2020). Senescent cells progressively accumulate in tissues and secrete large amounts of pro-inflammatory cytokines, chemokines, and proteases as SASP factors. These mediators not only cause direct local tissue injury but also propagate inflammatory signals systemically, thereby exacerbating the progression of inflammaging (Aurora and Veis, 2022). For example, in periodontitis models, aging may amplify local innate immune signaling and SASP-associated inflammatory responses, which in turn can exacerbate alveolar bone resorption. Toll-like receptor 9 (TLR9) may participate in this process under specific conditions, particularly when bacterial or mitochondrial DNA accumulates in inflamed tissues (Albuquerque-Souza et al., 2022). However, the relative contribution of TLR9 compared with TLR2 and TLR4 appears to be context dependent.In addition, gut microbiota dysbiosis is another critical contributor. Intestinal microbial imbalance can impair gut barrier integrity, allowing microbial products such as endotoxins to translocate into the circulation and thereby trigger systemic immune activation and chronic inflammation.This immune activation induced by gut dysbiosis is considered one of the major susceptibility factors in osteoporosis (Sapra et al., 2025). Adipose tissue, particularly abdominal fat redistributed and expanded with age, also plays an important role in inflammaging. Beyond serving as an energy storage organ, adipose tissue is an active endocrine organ capable of secreting a variety of adipokines and inflammatory mediators, including leptin, resistin, and adiponectin (Cao et al., 2020). During aging, adipose tissue undergoes functional alterations that increase the secretion of pro-inflammatory adipokines, further intensifying systemic low-grade inflammation and exerting adverse effects on bone metabolism (Cao et al., 2020). Collectively, inflammaging represents a complex pathophysiological state maintained by senescent cell-derived SASP, gut microbiota dysbiosis, and adipose tissue endocrine dysfunction, forming the core inflammatory basis of age-related osteoporosis.

### 5.2 Mechanisms by which chronic inflammation promotes bone resorption

Chronic inflammation is a major driver of bone resorption. Pro-inflammatory cytokines such as TNF-α, IL-1, and IL-6 promote osteoclast formation and also affect bone marrow stromal cells and osteoblasts (Ragipoglu et al., 2020). In inflammatory diseases such as rheumatoid arthritis (RA) and psoriatic arthritis (PsA), these cytokines increase RANKL and reduce OPG expression in stromal cells and osteoblasts, which shifts bone remodeling toward osteoclast activation (Orsolini et al., 2020; Swarnkar et al., 2021). Mast cells can also contribute by releasing osteoclastogenic mediators such as TNF and IL-6 in the bone microenvironment (Ragipoglu et al., 2020). Overall, inflammatory cytokines promote bone loss through both direct effects on osteoclast precursors and indirect effects on bone-forming cells.

NF-κB is a key signaling pathway linking inflammation to bone resorption. It is activated by RANKL through RANK and can also be stimulated by inflammatory cytokines and other stress signals (Swarnkar et al., 2021). NF-κB functions as a central inflammatory hub that couples cytokine signaling, oxidative stress, and cellular damage to skeletal remodeling. In the aging skeleton, mitochondrial dysfunction, ROS accumulation, telomere erosion, and declining DNA repair capacity progressively compromise genomic integrity, generating genotoxic stress. Persistent DNA lesions engage DNA damage response programs, including ATM/ATR-p53-p21 and p16-dependent pathways, thereby enforcing cell-cycle arrest, senescence, apoptosis, and loss of osteogenic competence. Senescent and damaged cells, in turn, amplify NF-κB-dependent inflammatory circuits and promote a pro-resorptive microenvironment that favors osteoclastogenesis. Thus, genotoxic stress may serve as a mechanistic axis linking metabolic dysfunction, senescence-associated inflammation, and age-related bone loss (Swarnkar et al., 2021). Once activated, NF-κB promotes osteoclast differentiation by inducing factors such as NFATc1 and also suppresses osteoblast activity. In wear particle-induced peri-implant osteolysis, persistent NF-κB activation increases osteoclast formation and reduces bone formation (Yu et al., 2020). Inhibition of miR-106b can reduce this process by blocking NF-κB activation (Yu et al., 2020). NF-κB also interacts with other pathways. In periodontitis, IL-1β and TNF-α promote osteoclast differentiation through NF-κB (Wang X. et al., 2021). In RA, JAK-STAT cooperates with NF-κB in inflammatory bone erosion, and agents such as tofacitinib may reduce bone loss by suppressing these pathways (Orsolini et al., 2020). Therefore, targeting NF-κB signaling and its upstream regulators represents an important therapeutic strategy for intervening in inflammation-induced bone resorption.

## 6 Direct effects of systemic metabolic dysregulation on the skeleton

### 6.1 Aberrant glucose metabolism and advanced glycation end products (AGEs)

During aging, abnormal glucose metabolism becomes an important factor in osteoporosis. Aging is often accompanied by insulin resistance and impaired glucose tolerance, and hyperglycemia has direct harmful effects on bone (Upadhyay and Kumar, 2025). Clinical studies in cystic fibrosis have shown that even early glucose abnormalities are associated with lower lumbar spine bone mineral density, and glycated hemoglobin is negatively related to bone density (Uçar et al., 2026). These findings suggest that hyperglycemia itself constitutes an independent threat to bone health. Mechanistically, a high-glucose environment can directly suppress osteoblast function and promote osteoblast apoptosis.*In vitro*, high glucose markedly reduces alkaline phosphatase activity and collagen type I induction in MC3T3-E1 osteoblasts, thereby impairing bone formation (Lee et al., 2020). It may also disturb osteoclast function, leading to uncoupling of bone formation and resorption and abnormal bone remodeling (Upadhyay and Kumar, 2025; Lee et al., 2020). Another important mechanism is the formation of AGEs. In aging and diabetes, glucose can react with long-lived bone proteins such as collagen type I, and AGEs gradually accumulate in bone tissue (LLabre et al., 2022). AGEs bind to RAGE and activate oxidative stress and chronic inflammation, including increased IL-6 and TNF-α, which further impair bone cell function (Lee et al., 2020; Liu T. et al., 2024; Shi et al., 2024). At the same time, AGEs can directly cross-link collagen fibers and damage the structure of the bone matrix, which reduces bone toughness and increases fracture risk (LLabre et al., 2022; Li G. et al., 2024). This helps explain the “diabetic bone paradox,” in which fracture risk is elevated even when bone mineral density is normal or increased (Li G. et al., 2024; Lee and Hwang, 2020). Clinical and experimental studies also support this mechanism, showing higher AGE levels in patients with low bone mass and increased AGE accumulation in diabetic animal models, which is associated with reduced bone toughness (Zawada et al., 2025). In addition, removal of glycation products can partially restore bone toughness *in vitro* (LLabre et al., 2022). *In vitro* removal of glycation products from bone tissue using phenylthiazolium chloride (PTC) partially restores the toughness of diabetic bone, directly demonstrating the critical role of AGEs in diabetic skeletal fragility (LLabre et al., 2022). Therefore, interventions that reduce AGE formation or block the AGE-RAGE pathway may help improve bone quality and lower fracture risk.

### 6.2 Dysregulated lipid metabolism and adipose endocrine function

During aging, dysregulation of lipid metabolism is another important pathophysiological factor contributing to osteoporosis. With advancing age, circulating free fatty acid (FFA) levels often increase, and abnormal accumulation of adipose tissue in the bone marrow becomes more pronounced, particularly in obesity and metabolic syndrome. Elevated circulating FFAs and excessive marrow fat deposition may induce lipotoxicity, thereby directly or indirectly impairing skeletal homeostasis (Zhou et al., 2026). In the bone marrow microenvironment, lipid overload can inhibit the osteogenic differentiation of BMSCs and shift them toward adipogenesis, reducing the osteoblast pool and increasing marrow fat. This imbalance represents an important cellular mechanism underlying osteoporosis (Zhou et al., 2026). Endoplasmic reticulum stress, a cellular response to the accumulation of misfolded proteins, is excessively activated under lipotoxic conditions and triggers the unfolded protein response (UPR); if stress persists, it ultimately leads to osteoblast apoptosis (Yu et al., 2024). At the same time, mitochondrial damage increases ROS production, promotes oxidative stress, and suppresses bone formation through pathways such as NF-κB (Cheng et al., 2024; Lin et al., 2025). High-fat diet models also show that abnormal lipid metabolism worsens the bone marrow microenvironment and weakens bone repair and remodeling capacity (Yang R. et al., 2025).

Adipose tissue is also an endocrine organ and affects bone through adipokines. Leptin mainly acts through the central nervous system and sympathetic signaling to inhibit osteoblast activity and promote bone resorption (Zhou et al., 2026).In contrast, the role of adiponectin in bone metabolism remains controversial and may be bidirectional. On the one hand, adiponectin is considered to exert anti-inflammatory and insulin-sensitizing effects, and may indirectly protect bone by improving the metabolic milieu; on the other hand, some studies suggest that adiponectin may also act directly on osteoblasts and osteoclasts, although its precise effects appear to depend on cell type, concentration, and the local microenvironment and remain to be fully clarified (Zhou et al., 2026). Resistin, by contrast, is generally regarded as a pro-inflammatory and pro-osteoclastogenic adipokine. Moreover, in the context of aging and obesity, adipose tissue dysfunction alters the adipokine secretion profile, characterized by enhanced leptin resistance, reduced adiponectin levels, and increased production of pro-inflammatory mediators such as resistin. This imbalance drives chronic inflammation and further aggravates bone metabolic disorders through immune modulation, including effects on macrophage M1/M2 polarization and Th17/Treg cell balance, thereby establishing a vicious cycle within the adipose-immune-bone axis that accelerates osteoporosis progression (Li et al., 2026).

### 6.3 Imbalance in energy and protein metabolism

During aging, imbalances in energy and protein metabolism constitute an important pathophysiological basis for osteoporosis. With advancing age, appetite often declines and digestive and absorptive capacity deteriorates, directly resulting in insufficient protein and total energy intake.Protein is a critical substrate for the synthesis of organic components in the bone matrix, such as collagen; inadequate intake directly impairs osteoblast synthetic function and leads to a shortage of amino acids required for bone matrix production (Long et al., 2025; Groenendijk et al., 2023). At the same time, insufficient energy intake places the body in an “energy crisis” state. To maintain essential physiological functions, limited energy may be preferentially allocated to more vital organs, while bone formation and repair are relegated to a secondary position, further weakening bone formation (Martyniak et al., 2021). Studies have shown that older adults, particularly elderly women, are at high risk of protein-energy malnutrition, and this nutritional status is significantly associated with reduced bone mineral density and increased fracture risk (Katage et al., 2025). This metabolic imbalance caused by both reduced intake and increased consumption leaves the skeleton in a prolonged state of net deficit, thereby laying the metabolic foundation for osteoporosis.

Muscle and bone are closely linked anatomically, functionally, and metabolically. Age-related loss of muscle mass, namely, sarcopenia, and bone loss, namely, osteoporosis, often occur concurrently, forming the so-called “osteosarcopenia” syndrome (Dowling et al., 2023). These two conditions share common pathological mechanisms, including aging, chronic low-grade inflammation, hormonal changes such as declines in sex steroids and growth hormone, and cellular senescence (Föger-Samwald et al., 2022). For example, senescent cells that accumulate during aging secrete large amounts of pro-inflammatory mediators, collectively referred to as the SASP, including IL-6 and TNF-α. These factors not only directly stimulate osteoclastogenesis and suppress osteoblast function, but also promote muscle protein degradation, leading to muscle atrophy (He et al., 2023; Sapra and Srivastava, 2025). Reduced muscle mass leads to diminished physical activity and decreased mechanical loading on the skeleton, thereby weakening the mechanical stimuli required to maintain bone mass (Crews, 2022). Conversely, skeletal fragility and pain caused by osteoporosis can further limit activity, exacerbating muscle disuse and atrophy and creating a vicious cycle. At the molecular level, key signaling pathways such as AMPK-mTOR play central roles in regulating cellular energy metabolism, autophagy, and senescence, and their dysregulation contributes to the pathogenesis of both sarcopenia and osteoporosis (Liu B. et al., 2024). Therefore, interventions targeting energy and protein metabolic imbalance as well as muscle-bone crosstalk, such as nutritional support to improve protein intake, pharmacological modulation of energy-sensing pathways using agents like metformin, or the application of senolytics to eliminate senescent cells, may represent effective strategies for preventing and treating age-related osteoporosis.

## 7 Integration and crosstalk of key signaling pathways

### 7.1 Aging-related alterations in core pathways regulating osteogenesis and osteoclastogenesis

During aging, the Wnt/β-catenin pathway is suppressed mainly because its endogenous inhibitors, especially sclerostin and Dkk1, are increased. This inhibits osteogenic differentiation of BMSCs and osteoblast activity, which contributes to the decline in bone formation in senile osteoporosis (SOP) (Föger-Samwald et al., 2022). In aged mouse BMSCs, activation of 4-1BB upregulates Dkk1 through the p38 MAPK pathway and further suppresses Wnt signaling (Wan et al., 2021). At the molecular level, this is associated with reduced nuclear translocation and transcriptional activity of β-catenin. Chronic oxidative stress also affects this pathway. In a conditional MACF1 knockout mouse model, oxidative stress increased FoxO1 expression and nuclear translocation, which enhanced FoxO1–β-catenin interaction and reduced β-catenin binding to TCF7, thereby impairing osteoblast differentiation (Su et al., 2026). Transcriptomic analyses of elderly osteoporotic patients also showed enrichment of Wnt-related genes, supporting the importance of this pathway in SOP (Wu et al., 2023). Overall, aging inhibits Wnt/β-catenin signaling by increasing endogenous antagonists and altering β-catenin-associated transcriptional regulation.The RANK/RANKL/OPG axis is the main regulator of osteoclastogenesis and bone resorption. During aging and inflammation, RANKL tends to increase, whereas OPG is relatively insufficient, shifting the balance toward osteoclast formation. Aging BMSCs often show reduced osteogenic capacity and increased adipogenic differentiation, which may further disturb the OPG/RANKL ratio (Qadir et al., 2020). Aging can also act directly on osteoclast precursors. In high-passage RAW264.7 cells, membrane RANK expression decreases with serial passaging, likely due to hypermethylation of CpG sites in the RANK promoter (Kitazawa et al., 2024). This reduces responsiveness to soluble RANKL and impairs osteoclast differentiation, consistent with the low-turnover phenotype seen in SOP. However, *in vivo* aging is also accompanied by chronic inflammation. TNF-α can stimulate osteoblasts and stromal cells to produce RANKL, and senescent cells with their SASP may further promote osteoclastogenesis (Pignolo et al., 2025). Thus, aging affects this axis in two ways: it weakens osteoclast precursor sensitivity to RANKL while increasing RANKL availability and possibly lowering OPG production. The overall result is disruption of bone remodeling and age-related bone loss.

### 7.2 Roles of metabolic and stress-sensing pathways

The AMPK/SIRT1/PGC-1α axis is an important regulator of cellular energy balance and metabolic homeostasis. Its activity decreases with aging and this is associated with osteoporosis. AMPK acts as an energy sensor and is activated under energy stress, where it regulates downstream metabolic processes. Activation of AMPK can improve mitochondrial function and reduce oxidative stress, both of which are important for bone cell survival. In an iron overload-induced bone loss model, corylin suppressed Akt signaling and increased the nuclear translocation of FoxO1 and Nrf2, which enhanced antioxidant defense and mitochondrial function, indirectly supporting the role of upstream AMPK-related signaling (Zhang et al., 2019). SIRT1 is a NAD + -dependent deacetylase that is closely related to cellular metabolism and aging. Its activation promotes osteogenic differentiation, partly by deacetylating and activating PGC-1α, which supports mitochondrial biogenesis and function. Autophagy is also activated during osteoblast differentiation and is linked to mitochondrial quality control, suggesting that the AMPK/SIRT1 pathway may regulate osteogenesis through autophagy (Xu et al., 2020). In diabetic osteoporosis, Exendin-4 promotes osteogenesis by downregulating HDAC1 and activating Wnt/β-catenin signaling, while SIRT1 may also interact with HDAC1 to regulate osteogenesis-related genes (Deng et al., 2021). Overall, AMPK and SIRT1 help maintain energy balance and redox homeostasis, and they may support bone formation while limiting bone loss in age-related osteoporosis.

The PI3K/Akt/mTOR pathway integrates growth factor, nutrient, and metabolic signals. Its overactivation is associated with cellular senescence, autophagy inhibition, and osteoporosis. In aging, persistent mTOR activation suppresses autophagy, leading to the accumulation of damaged proteins and organelles and promoting bone cell dysfunction. In osteoblasts, TNF-α inhibits autophagy and downregulates Wnt/β-catenin signaling, which impairs osteogenic differentiation; autophagy inducers can reverse these effects (Chen et al., 2020). In glucocorticoid-induced osteoporosis, glucocorticoids may activate Akt-mTOR signaling, suppress autophagy, and promote osteocyte senescence. This pathway also contributes to osteoclast differentiation. Berberine inhibits RANKL-induced PI3K/Akt/NFATc1 signaling and reduces osteoclast formation and bone resorption (Zhou L. et al., 2020),while cytochalasin suppresses osteoclastogenesis by blocking RANKL-triggered PI3K/Akt, MAPK, and NF-κB signaling (Qian et al., 2020). In iron overload models, corylin protects against bone damage partly by inhibiting Akt-FoxO1 signaling and reducing oxidative injury (Zhang et al., 2019). Overall, the PI3K/Akt/mTOR pathway links external stress signals to bone cell fate, and moderate inhibition, especially of mTOR, may help restore autophagy and rebalance bone remodeling (Table 2).

**TABLE 2:** Crosstalk among key signaling pathways regulating osteogenesis, osteoclastogenesis, and metabolic homeostasis in age-related osteoporosis.

Category	Pathway	Core mechanism	Functional outcome/Representative interventions
Pro-osteogenesis	Wnt/β-catenin	Aging suppresses Wnt signaling mainly through upregulation of endogenous inhibitors such as sclerostin and Dkk1. Oxidative stress further enhances FoxO1–β-catenin interaction, weakening β-catenin binding to TCF7/TCF4 and reducing canonical Wnt transcriptional activity (He et al., 2023)	Impairs BMSC and osteoblast differentiation, leading to reduced bone formation and senile osteoporosis
	AMPK/SIRT1/PGC-1α	This axis senses cellular energy status and is downregulated during aging. Activation of AMPK or SIRT1 improves mitochondrial function, reduces oxidative stress, and promotes osteogenic differentiation *via* PGC-1α (Wu et al., 2023)	Enhances osteogenesis and maintains mitochondrial homeostasis. Representative interventions: corylin, Exendin-4
	HDAC1/Wnt interaction	In diabetic osteoporosis, Exendin-4 downregulates HDAC1 and activates Wnt/β-catenin signaling. SIRT1 may interact with or antagonize HDAC1 to regulate osteogenesis-related gene expression (Kitazawa et al., 2024)	Promotes osteogenesis and improves metabolic osteoporosis
Pro-osteoclastogenesis	RANK/RANKL/OPG	Aging and inflammation increase RANKL availability while OPG becomes insufficient, shifting bone remodeling toward osteoclastogenesis. In some aging models, epigenetic silencing of the RANK promoter reduces precursor responsiveness to RANKL (Crews, 2022)	Dysregulates bone remodeling and contributes to net bone loss
	PI3K/Akt/mTOR	Persistent mTOR activation suppresses autophagy and accelerates bone cell senescence. This pathway also contributes to RANKL-induced osteoclast differentiation (Zhang et al., 2019)	Promotes bone cell senescence, inhibits osteogenesis, and enhances osteoclastogenesis. Representative interventions: berberine, cytochalasin, corylin
Metabolism/Autophagy	AMPK/SIRT1/autophagy axis	SIRT1 deacetylates and activates PGC-1α and is closely linked to autophagy and mitochondrial quality control. The AMPK/SIRT1 pathway may regulate osteogenesis through autophagic mechanisms (Qadir et al., 2020)	Supports osteogenic differentiation and cell survival
	TNF-α–Autophagy–Wnt	TNF-α inhibits autophagy and downregulates Wnt/β-catenin signaling in osteoblasts, thereby impairing osteogenic differentiation. Autophagy inducers can reverse these effects	Represents an important mechanism of inflammation-induced bone loss
Metabolism/Oxidative stress	FoxO1/Nrf2/Akt	In iron-overload-induced oxidative injury, corylin suppresses Akt signaling and enhances nuclear translocation of FoxO1 and Nrf2, strengthening antioxidant defense (Wu et al., 2023)	Attenuates oxidative injury and protects bone cell function

## 8 Therapeutic strategies: established osteoporosis treatments and emerging geroscience-based interventions

### 8.1 Established pharmacological therapies for osteoporosis

Current evidence-based therapies for osteoporosis are mainly classified into antiresorptive and anabolic agents. Antiresorptive drugs, including bisphosphonates and denosumab, reduce bone resorption by suppressing osteoclast activity, differentiation, or survival. Bisphosphonates bind to mineralized bone matrix and are internalized by osteoclasts during bone resorption, leading to osteoclast dysfunction or apoptosis. Denosumab, a monoclonal antibody against receptor activator of nuclear factor-κB ligand (RANKL), prevents osteoclast formation, activation, and survival. These agents have demonstrated efficacy in reducing fracture risk and remain first-line options for many patients with postmenopausal or age-related osteoporosis.

Anabolic therapies include teriparatide, abaloparatide, and romosozumab. Teriparatide and abaloparatide are parathyroid hormone receptor agonists that stimulate bone formation when administered intermittently. Romosozumab, an anti-sclerostin antibody, enhances Wnt/β-catenin signaling, thereby increasing bone formation and simultaneously reducing bone resorption. These anabolic agents are particularly valuable for patients with severe osteoporosis, very high fracture risk, or inadequate response to antiresorptive therapy.

Although approved therapies effectively increase bone mineral density and reduce fracture risk, they do not fully reverse the upstream aging-related mechanisms that contribute to skeletal fragility, including cellular senescence, mitochondrial dysfunction, inflammaging, marrow adiposity, impaired proteostasis, and systemic metabolic dysregulation. Therefore, emerging geroscience-based interventions should currently be viewed as potential complementary strategies rather than replacements for established osteoporosis therapies.

### 8.2 Targeted elimination of senescent cells: Senolytics

Cellular senescence is increasingly recognized as an upstream driver of skeletal aging. Senescent osteoblast-lineage cells, osteocytes, immune cells, and bone marrow stromal cells can accumulate with age and secrete a SASP, thereby promoting chronic inflammation, osteoclastogenesis, impaired osteogenesis, and marrow adiposity. Senolytics are a class of agents designed to selectively eliminate senescent cells, with the combination of dasatinib plus quercetin (D + Q) being one of the most extensively studied approaches.Preclinical studies have shown that senolytics reduce senescent cell burden in bone, suppress SASP-related inflammatory factors, and improve bone mass, bone strength, and marrow adiposity (Khosla et al., 2020). In aging and radiation-induced osteoporosis mouse models, D + Q reduced senescence markers such as p16Ink4a and p21, decreased SASP-associated mediators, and improved bone microarchitecture (Chandra et al., 2020). Additional studies suggest that senescent cell accumulation contributes to age-related bone loss and bone marrow adipose tissue expansion, and that D + Q can partially reverse these phenotypes (Chandra et al., 2022a). Quercetin alone has also been reported to clear senescent bone marrow mesenchymal stromal cells, restore proliferative and osteogenic capacity, and reduce adipogenic differentiation (Zhang et al., 2020). Notably, some evidence indicates that eliminating p21-positive senescent cells may be more effective than targeting p16-positive cells in radiation-induced osteoporosis and marrow adiposity, highlighting the heterogeneity of senescent cell populations in bone (Chandra et al., 2022b).

Beyond local skeletal effects, senolytics may attenuate systemic inflammaging, which is relevant because senescent cells and their SASP contribute to multiple age-related disorders, including osteoporosis, frailty, metabolic syndrome, type 2 diabetes, and cardiovascular disease (Aurora and Veis, 2022; Kaur and Farr, 2020). The field is moving from animal studies toward early clinical testing, and there is interest in their use for osteoporosis, metabolic syndrome, and type 2 diabetes (Khosla et al., 2020). However, clinical translation remains at an early stage. Major challenges include limited bioavailability, potential off-target toxicity, uncertainty regarding optimal treatment windows, and the need for biomarkers that identify patients most likely to benefit (Xing et al., 2023). To improve specificity, bone-targeted delivery platforms are being developed, including quercetin-loaded liposomes modified with bone-affinity peptides and small extracellular vesicles carrying β-galactosidase-activated prodrugs (He et al., 2024). These strategies may enable more selective clearance of senescent cells in the bone microenvironment while minimizing systemic toxicity.

### 8.3 Metabolic reprogramming and drug repurposing

Aging-related osteoporosis is accompanied by profound metabolic remodeling in bone cells and their surrounding microenvironment. Metabolic reprogramming refers to adaptive or maladaptive changes in cellular energy utilization, mitochondrial function, biosynthesis, redox balance, and nutrient-sensing pathways. In aging bone, mitochondrial dysfunction, oxidative stress, impaired autophagy, and altered AMPK–mTOR signaling contribute to osteoblast dysfunction, osteocyte senescence, osteoclast activation, and marrow adipogenesis. Because senescent cells remain metabolically active despite permanent cell-cycle arrest, targeting their altered metabolic state provides a rationale for therapeutic intervention (Kim et al., 2024). Drug repurposing is particularly attractive in this context because many metabolic drugs have established safety profiles, defined pharmacokinetics, and relatively low development costs (Chaudhary et al., 2025). Metformin has attracted attention for its potential anti-aging effects beyond glycemic control. Mechanistically, metformin activates AMP-activated protein kinase (AMPK), suppresses mTOR signaling, improves mitochondrial homeostasis, and may reduce oxidative stress and inflammatory activation (Chaudhary et al., 2025). Since mTOR overactivation is associated with cellular senescence, impaired autophagy, and reduced stress resilience, metformin may help restore autophagic flux and limit the accumulation of damaged proteins and organelles (Kim et al., 2024). These effects could be beneficial in aging bone by improving osteoblast function, reducing senescence-associated inflammation, and modulating osteoclast activity.

Rapamycin and related mTOR inhibitors represent another class of geroscience-based candidates. By inhibiting mTOR complex 1, rapamycin promotes autophagy, enhances proteostasis, reduces cellular damage, and may limit SASP-associated inflammatory signaling (Sun et al., 2026). In preclinical studies, rapamycin has shown beneficial effects on lifespan and several age-related tissue phenotypes, including skeletal outcomes. In the context of senile osteoporosis, rapamycin may improve bone homeostasis by reducing cellular senescence, restoring autophagic activity, and shifting bone-cell metabolism toward a less inflammatory and less catabolic state. Nevertheless, the clinical use of mTOR inhibition for osteoporosis remains exploratory, and potential adverse effects, dose optimization, treatment duration, and patient selection require careful evaluation.

### 8.4 Nutritional, exercise, and microbiome-based interventions

Non-pharmacological interventions remain essential components of osteoporosis prevention and management, particularly in older adults with metabolic dysfunction, frailty, or sarcopenia. Adequate protein and energy intake, together with supplementation of calcium, vitamin D, and selected antioxidants, can support musculoskeletal health. Aging is frequently accompanied by reduced appetite, impaired nutrient absorption, chronic inflammation, and anabolic resistance, all of which increase the risk of protein-energy malnutrition and contribute to both sarcopenia and osteoporosis (Buettmann et al., 2022). Sufficient protein intake, especially leucine-rich protein, helps preserve muscle mass and function. Because muscle and bone are mechanically and biochemically coupled, sarcopenia reduces skeletal loading and may accelerate bone loss (Jung et al., 2021). Calcium and vitamin D remain standard supportive measures for osteoporosis prevention and treatment. Vitamin D enhances intestinal calcium absorption and also exerts direct effects on bone cells through the vitamin D receptor. In addition, oxidative stress increases with age and contributes to osteoblast dysfunction, osteocyte damage, and osteoclast activation. In patients with osteoporosis, oxidative stress markers are often elevated, whereas antioxidant enzyme activity is reduced (Ahuja et al., 2022). Antioxidants such as N-acetylcysteine (NAC) may therefore help restore redox balance, although their clinical efficacy in osteoporosis requires further validation.

Exercise is another cornerstone intervention for age-related skeletal fragility. Combined resistance and aerobic exercise can enhance muscle mass and strength, stimulate bone formation through mechanical loading, and improve systemic metabolism and inflammatory tone. Disuse and aging share overlapping skeletal features, including increased bone resorption, reduced bone formation, and expanded marrow adiposity (Buettmann et al., 2022). Resistance training activates mechanotransduction pathways, including Wnt/β-catenin signaling, and promotes osteoblast differentiation and bone formation (Johnson De Sousa Brito et al., 2021). Exercise also improves skeletal muscle quality and function, thereby maintaining mechanical support and potentially modulating bone through myokines (Jung et al., 2021). Aerobic exercise further improves insulin sensitivity, mitochondrial function, and chronic low-grade inflammation, which are closely linked to skeletal aging (Anagnostis et al., 2022). It may also improve mitochondrial function, reduce ROS accumulation, and delay senescent cell accumulation in the bone microenvironment (Doolittle et al., 2021). Overall, combined exercise has effects on both muscle and bone and is a practical strategy for reducing age-related skeletal fragility.

The gut–bone axis has also emerged as a promising target. The gut microbiota connects nutrition, immune regulation, metabolism, and skeletal remodeling, whereas dysbiosis has been associated with osteoporosis. Probiotics, prebiotics, and postbiotics may support bone health by improving calcium absorption, strengthening intestinal barrier function, reducing systemic inflammation, and modulating immune responses. In glucocorticoid-induced osteoporotic mice, reduced *Lactobacillus* abundance has been reported, whereas bovine colostrum-derived exosomes improved bone mineral density and restored gut microbial composition (Yun et al., 2020). During aging, gut barrier dysfunction may promote endotoxin translocation and chronic activation of inflammatory pathways such as NF-κB, thereby enhancing osteoclastogenesis and bone resorption (Buettmann et al., 2022). Microbial metabolites, including short-chain fatty acids such as butyrate, can exert anti-inflammatory and immunomodulatory effects and may influence bone remodeling. Some probiotic strains may also promote vitamin K production or improve mineral absorption, further supporting skeletal health. In addition to systemic gut-derived influences, local marrow aging may interact with metabolic and inflammatory signals. For example, senescent bone marrow adipocytes have been reported to acquire an SASP-like phenotype and secrete serum amyloid P component, contributing to skeletal amyloidosis and bone loss (Kumar et al., 2025). Although this finding is not directly a gut microbiota mechanism, it illustrates how local adipose aging, systemic inflammation, and metabolic dysfunction may converge to impair bone homeostasis. Collectively, nutritional optimization, exercise, and microbiome-based approaches may complement pharmacological therapy by targeting modifiable systemic drivers of skeletal aging.

## 9 Conclusion

The pathogenesis of senile osteoporosis is no longer explained only by sex hormone deficiency. It is now understood as a process involving changes at the cellular, metabolic, and systemic levels. BMSC senescence, lineage shift, SASP, mitochondrial dysfunction, chronic low-grade inflammation, and abnormal glucose and lipid metabolism are closely linked. These changes interact through pathways such as RANKL/OPG, Wnt/β-catenin, AMPK/SIRT1, and NF-κB, leading to an imbalance between bone formation and bone resorption. This also explains why antiresorptive drugs can slow bone loss but cannot fully restore bone structure or prevent fragility in older patients.

Future treatment will likely depend on combining local and systemic approaches. One direction is to target the bone microenvironment, for example, by removing senescent cells with senolytics or suppressing SASP. Another is to improve systemic metabolism through drugs such as metformin or through lifestyle interventions that activate the AMPK/SIRT1 axis. These strategies may work better together than alone. Improving mitochondrial function, for example, can reduce oxidative stress, delay senescence, and support osteoblast energy metabolism. Because of this, the most promising strategy is probably a multi-target and sequential combination approach, including senolytics, metabolic regulators, lifestyle intervention, and possibly gut microbiota-based therapies.

This shift also creates new demands for research and clinical practice. On the research side, systems biology approaches are needed to study how metabolism, inflammation, and the skeletal microenvironment interact. Better animal models are also needed to reflect human skeletal aging more accurately. On the clinical side, safety and efficacy must be confirmed in well-designed trials before these strategies can be widely used. Bridging basic mechanisms with clinical application will be essential for slowing skeletal aging and improving outcomes in elderly patients with osteoporosis.
